# Strength and Aerobic Physical Exercises Are Able to Increase Survival of *Toxoplasma gondii*-Infected C57BL/6 Mice by Interfering in the IFN-γ Expression

**DOI:** 10.3389/fphys.2016.00641

**Published:** 2016-12-23

**Authors:** Miguel J. S. Bortolini, Murilo V. Silva, Fábio M. Alonso, Luciana A. Medeiros, Fernando R. Carvalho, Lourenço F. Costa, Neide M. Silva, Nilson Penha-Silva, Tiago W. P. Mineo, José R. Mineo

**Affiliations:** ^1^Laboratório de Imunoparasitologia, Instituto de Ciências Biomédicas, Universidade Federal de UberlândiaUberlândia, Brazil; ^2^Laboratório de Exercícios Físicos Resistidos e Aeróbicos, Centro de Ciências da Saúde e do Desporto, Universidade Federal do AcreRio Branco, Brazil; ^3^Laboratório de Imunopatologia, Instituto de Ciências BiomédicasUberlândia, Brazil; ^4^Laboratório de Biofisicoquímica, Instituto de Genética e BioquímicaUberlândia, Brazil

**Keywords:** strength and aerobic exercise, *Toxoplasma gondii*, murine model, immune response, parasite burden, IFN-γ levels, IFN-γ/IL10 ratios

## Abstract

Physical exercise has been implicated in several immunophysiological improvements, particularly during the aging process, when an immunocompromised status could be established. *Toxoplasma gondii* is a protozoan parasite that causes a widespread opportunistic infection, which may present severe consequences, mainly to the fetus and immunocompromised patients. It is estimated that one-third of the human population worldwide has been infected by this parasite, being the reactivation during immunesenescence an unexplored public health issue. The major purpose of the present study was to observe the immunophysiological differences between exercised vs. sedentary C57BL/6 male mice that have been experimentally infected by *T. gondii*. In the first set of experiments, the animals were infected after exercising and three groups were set up: experimental groups—infected sedentary (IS, *n* = 6); infected exercised (IEx, *n* = 6) and control group—non-infected sedentary (NIS, *n* = 6). When stimulated *in vitro* by *T. gondii*-soluble tachyzoite antigen, it was found that splenocytes from exercised group produced higher levels of IFN-γ, as well as of IFN-γ/IL-10 ratios in comparison with splenocytes from sedentary animals (*P* < 0.001). However, it was not found significant differences concerning quantification of *T. gondii* genomic DNA by qRT-PCR and immunohistochemistry analysis in brain cysts from both group of animals (*P* > 0.05). In order to further investigate the consequences of these data for the host, a second set of experiments was performed, when the animals were infected before exercising and four groups of animals were established for comparison purpose, as follows: experimental groups—infected sedentary (IS, *n* = 7); infected exercised (IEx, *n* = 6) and control groups—non-infected sedentary (NIS, *n* = 6) and non-infected exercised (NIEx, *n* = 6). It was found significant differences in the survival rates of the exercised group the animals, as they survived longer than sedentary groups (*P* = 0.0005). In both sets of experiments, mice have been submitted to moderate exercises: aerobic (14 m/min; 3 x/week) and strength (60–80% of one maximum repetition; 2 x/week). Overall, our findings are showing that the aerobic and strength exercises are able to modulate immune response against *T. gondii* infection, being these immunological features beneficial to the host.

## Introduction

Exercise immunology has emerged as a new field within the immunology science since the 80s, although landmark findings had been reported as early as 1893 (Shephard, 2010). Nowadays, this expression can be defined as the field of the biomedical science that studies the interference of physical exercise in both induction and regulation of immune response and that is why exercise immunology is an essential subject for both physical education and immunology communities (Bortolini et al., 2013). Several studies concerning physical exercise found plenty improvements in immune system (Walsh et al., 2011a,b) and recent experimental works have reinforce its importance during various processes, as follows: (a) to fight cancer (Almeida et al., 2009), or protect graft (Fiuza-Luces et al., 2013) (b) to improve the clearance of pathogens, e.g., *Trypanosoma cruzi* (Schebeleski-Soares et al., 2009; Moreira et al., 2014); and (c) to promote regulation of cytokines as IFN-γ, TNF, TGF-β1, IL-4, IL-10, and IL-12 during infection by *Leishmania* (Terra et al., 2013), as well as regulation of TNF (Chao et al., 1992) or TNF and TGF-β (Moreira et al., 2014), during *Toxoplasma gondii* and *T. cruzi* infection, respectively.

*T. gondii* is a widespread opportunist parasite that is estimated to infect one-third of the human population worldwide (Weiss and Dubey, 2009). It causes toxoplasmosis which is important in congenital infection and in immunosuppressed reactivation (Desmonts and Couvreur, 1974; Leser et al., 2003) as in HIV/AIDS (Saadatnia and Golkar, 2012). In healthy people it is associated with ocular pathologies (Roberts et al., 2001). Recently, it has been associated with schizophrenia (Prandovszky et al., 2011; McConkey et al., 2013). For immunocompetent people, toxoplasmosis is most asymptomatic and, when the infection by *T. gondii* becomes symptomatic, the clinical manifestations are characterized as unspecific (Montoya et al., 2004), though ocular or neurological complications can be present (Luft et al., 1993). The aging process leads to the losing of function in most systems (e.g., immunological system), and *T. gondii* reactivation becomes potential in this period (Gardner and Remington, 1978a,b). In this context, a competent immune system is fundamental to avoid *T. gondii* reactivation (Saadatnia and Golkar, 2012).

To the best of our knowledge, there is only one study in the literature that has assessed the effects of the experimental *T. gondii* infection during exercise (Chao et al., 1992). Although the World Health Organization (2010) recommends strength and aerobic physical activities for adults, so far there is no study approaching the effects of strength exercise lonely or combined with aerobic one during infection with *T. gondii* or other parasite. In this scenario, our study was designed to assess the immunophysiological differences between exercised vs. sedentary C57BL/6 male mice that have been infected by *T. gondii*.

## Materials and methods

### Animals

C57BL/6 Male mice were housed in cages (30 × 20 × 13 cm; 6–7 per cage) and kept under standard conditions, natural photo-period (from 6:00 a.m. to 6:00 p.m.), in a temperature-controlled room (25 ± 2°C), with *ad libitum* food and water intake, in the Animal Facility Center from Federal University of Uberlândia, Brazil. The experimental procedures were conducted according to the institutional guidelines and approved by the Ethical Committee in Animal Experimentation (CEUA-UFU Protocol N° 053/10). The physical exercises were conducted at the dark cycle from 07:00 p.m. to 10:00 p.m. Mice had previous 1 week adaptation in experimental room and exercises.

### Experimental groups

**Figure 1:**
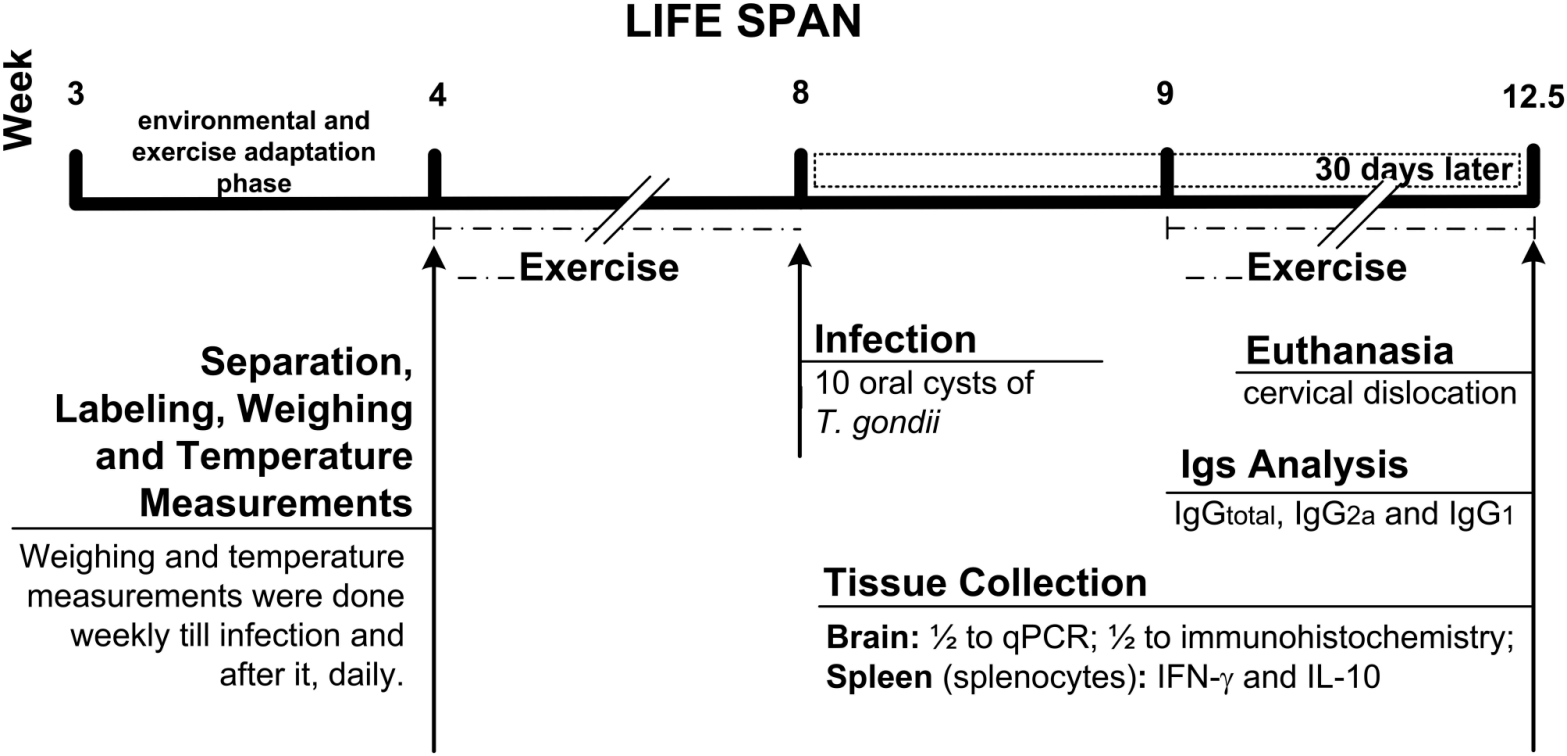
**Life span scheme of the first set of experiments**.

Two experimental groups were designed in the present study to evaluate the effect of exercise during acute or chronic phase of infection by *T. gondii* ME49 strain, as follows: (i) the first set of experiments was set up by infection with 10 parasite brain cysts and 18 C57BL/6 male mice 3 week-old were randomly placed in three different groups: control group—non-infected sedentary (NIS; *n* = 6); and experimental groups—infected sedentary (IS; *n* = 6); infected exercised (IEx; *n* = 6). To this set, mice were previously exercised (4–8- and 9–12.5-week-old; see scheme in Figure 1); (ii) in the second set of experiments, 25 C57BL/6 male mice 5 weeks old were allocated randomly in four of the following groups: control groups—non-infected sedentary (NIS, *n* = 6) and non-infected exercised (NIEx, *n* = 6); experimental groups: infected sedentary (IS, *n* = 7); and infected exercised (IEx, *n* = 6). To this second set, mice were previously infected with five parasite brain cysts and then chronically exercised (from 11 to 25 weeks old; see scheme in Figure 8).

### Aerobic exercise

All exercised mice were submitted to 1 week period for adaptation phase using a treadmill mechanically modified to work at low speed for animal exercising (Cardiofit, Movement, Brazil; Figure 12, See also Figures 1, 8). They had three running sessions per week (Mondays, Wednesdays, and Fridays) at 14 m/min speed (moderated speed), which have been progressively increased their running time till the end of both sets of experiments. Session 1: mice ran eight times, 1 min recovery interval to each 2 min running; session 2: mice ran eight times, 1 min recovery interval to each 3 min running; session 3: mice ran six times, 1 min recovery interval to each 4 min running; from this session onwards, the mice ran six times, 1 min recovery interval to each 5 min running. The animals performed voluntary running and when necessary they were stimulated with slight touching using paper sheet to avoid injury.

### Resistance exercise

The resistance exercise consisted in two sessions per week (Tuesdays and Thursdays) using our own built climbing apparatus (Figure 13) and the entire whole apparatus, which allows one to control the load for strength exercise (Figure 14), except the lead balls (weight) in adaptation phase (Figures 1, 8). Step one: before starting the exercise, one-repetition maximum strength (1-RM) were taken from all the exercised mice. Step two: mice started practice loading 60% of 1-RM in the first session and it was increased progressively till reach 80% to the next sessions. Step three: when they reach 80% or performed more than three straight sessions, they had their 1-RM taken again and kept the same load. After infection, they could have their weights decreased. During each session, they climbed 12 times with 3–4 min recovery interval. Mice were positioned at the bottom of the climbing apparatus and the repetitions were valid when the mouse performed 14–15″ climbing to the top or staying inert (without falling). If mouse fell, the repetition was not count and he should perform an extra climbing for each missed one at the end of the section.

### Weight and temperature measurement

Mice from the both sets of experiments had their weight determined (AUW220D, Shimadzu, Philippines) and a digital thermometer (TCI1000, Avita, Wujiang Co., LTD, China) was used to measure temperature. These procedures were carried out between 6:00 p.m. to 7:00 p.m. weekly till infection and daily after it.

### Parasites

Brain cysts of *T. gondii* ME49 strain were obtained from *Calomys callosus* after 30–45 days of infection, as described previously (Barbosa et al., 2007). Briefly, brains were removed, homogenized, washed in sterile phosphate-buffered saline (PBS, pH 7.2), centrifuged at 1000 × g for 10 min and cysts were counted under light microscopy (40x magnification). Brain tissue containing the cysts was homogenized in saline and the suspensions were adjusted to contain 5 or 10 cysts in each 100 μl aliquot. Just after the infection (100 μl aliquot containing cysts), by using the same gavage needle and syringe, it was given to them 100 μl more of the water used to hydrated them regularly. Infection procedures were carried out perorally (PO). In the first set of experiments, 8 week-old mice were infected with 10 cyst/100 μl (see scheme in Figure 1). In the second set of experiments, 6 week-old mice were infected with 5 cysts/100 μl (see scheme in Figure 8).

### Seroconversion assay

To confirm the seroconversion of the mice to *T. gondii* in both sets of experiments, an indirect ELISA to detect serum IgG antibodies to *T. gondii* was carried out before and after the infection, as previously described (Barbosa et al., 2007). To detect the IgG subclasses, an indirect ELISA also were carried out to analyze IgM (only for animals from the second set of experiments), IgG_1_ and IgG_2a_, as previously described (Kang et al., 2006).

### Cytokines profile analysis

To the first set of experiments, spleens were macerated in RPMI medium and cell suspensions were filtered, washed with medium, treated with lysis buffer (0.16 M NH_4_Cl and 0.17 M Tris–HCl, pH 7.5), washed again and suspended in RPMI medium containing 10% CFS. Cells (2 × 10^5^ cells/200 μL/well) were cultured in triplicate in 96-well-culture plates in the presence of mitogen (Concanavalin A—ConA, 10 ng/ml from Sigma-Aldrich Chemical Co., St. Louis, MO, US), Lipopolysaccharide from *Salmonella enterica*—serotype enteritidis (LPS; 1 μg/ml from Sigma-Aldrich Chemical Co., St. Louis, MO, US), *T. gondii*-soluble tachyzoite antigen (STAg; 50, 25, and 10 μg/ml) or medium alone and incubated at 37°C in 5% CO_2_ atmosphere. After 72 h, the plate was centrifuged (400 × g, 4 min, 4°C), the cell-free supernatants were collected and stored at −70°C for cytokine quantification. IL-10 and IFN-γ measurements were performed by sandwich ELISA kits according to manufacturer's instructions (BD Biosciences and R&D Systems). The reaction was revealed with peroxidase-streptavidin conjugate, followed by the addition of substrate containing hydrogen peroxide and TMB as a chromogen (BD Biosciences). Microplates were read in a plate reader (M2e, Molecular Devices, Sunnyvale, CA, USA) at 450 nm. The limit of detection was 31 pg/mL for both cytokines.

### Soluble antigen from *T. gondii*

The extraction of soluble tachyzoite antigen from *T. gondii* (STAg) was obtained as previously described (Mineo et al., 1980), with modifications. Briefly, pellets containing ~2 × 10^9^ tachyzoites from RH strain were suspended and washed twice in phosphate-buffered saline (PBS) at 720 g for 10 min at 4°C. Parasite suspensions were adjusted to 1 × 10^8^ tachyzoites/ml, treated with protease inhibitors (10 μg/ml aprotinin, 50 μg/ml leupeptin, and 1.6 mM phenylmethylsulfonylfluoride; all them from Sigma-Aldrich Chemical Co., St. Louis, MO, USA) and then lysed by five freeze- thaw cycles and further by sonication (six 60-Hz cycles for 1 min each) on ice. After centrifugation (10,000 g, 30 min, 4°C), supernatants were collected and filtered through a 0.2 μm membrane (Corning Costar Corp., Cambridge, USA). The protein concentration was determined by using the Lowry method (Lowry et al., 1951) and STAg aliquots were stored at −80°C.

### Quantitative real-time PCR

Brain parasite burden in mice was determined by quantitative real-time PCR (qRT-PCR), as previously described (Wahab et al., 2010). The primers were engineered to detect the repetitive area of 529 bp to *T. gondii*, using SYBR green system (Invitrogen, San Francisco, CA). The DNA extraction (from the right cerebral hemisphere) was carried out using Wizard SV Genomic DNA kit (Promega Co., Madison, WI), by taking macerated brain sample (20 μg) following manufacturer's instructions. The total DNA concentration was detected by UV spectrophotometry (260 nm) adjusting the concentration of DNA samples to 200 ng/μl. The assays to determine the *T. gondii* quantification were performed using “StepOnePlus™ PCR Systems” (Applied Biosystems, Foster City, CA), with Master Mix (Promega Co., Madison, WI), with 900 nM of each primer. All reactions were conducted simultaneously with the positive control samples carrying out a standard curve following seven 10-fold dilutions, starting from 1:10 containing 100 ng of parasite DNA. The parasite quantifications were counted by interpolation from a standard curve (10^−2^ a 10^−7^ ng), with equivalent of *T. gondii* DNA.

### Immunohistochemical analysis

Brain tissue parasitism was also determined by immunohistochemistry, as previously described (Silva et al., 2002). Tissue sections were incubated with 3% hydrogen peroxide, followed by 0.2 M citrate buffer (pH 6.0) for a 7-min cycle in microwaves to rescue antigenic sites. Next, sections were blocked with 2% non-immune goat serum and incubated with primary antibody (pooled sera from mice experimentally infected with *T. gondii*), diluted 1:100 in PBS plus 1% bovine serum albumin. As negative controls, paired tissue sections were incubated in the absence of primary antibodies (diluent only) or with non-immune mouse serum. Subsequently, secondary biotinylated goat anti-mouse IgG antibody (1:300; Sigma) and the streptavidin-biotinylated peroxidase complex (1:250; DAKO Corporation, Carpinteria, USA) were added and the reactions were developed with 0.03% H_2_O_2_ and 3,3-diaminobenzidine tetrahydrochloride (DAB; Sigma). Slides were counterstained with Harris haematoxylin and examined under light microscopy. Negative controls included brain tissue from non-immunized and unchallenged mice. Cysts and total parasites from the left cerebral hemisphere were determined by two independent investigators, under light microscopy using a 40 × objective.

### Statistical analysis

Kolmogorov-Smirnov test was used to assess normality distribution of the samples. The simultaneous comparisons among all groups were performed by ANOVA, followed by Bonferroni's multiple comparisons post-tests to examine all possible pair wise comparisons. The comparisons between SI and ExI groups by immunohistochemistry and qRT-PCR were done by Student *t*-test. The Kaplan-Meier test was applied to estimate the percentage of mice surviving at each time point after challenge and survival curves were compared using the Log-rank test. Values were expressed as mean ± *SD* and the differences were considered statistically significant when *P* < 0.05. All tests were carried out using GraphPad Prism 5.0 (GraphPad Software Inc., San Diego, USA).

## Results

All analyses were performed in seropositive mice for *T. gondii* (Figures 3A–G; except IgM for the first set of experiments). Concerning the first set of experiments, two mice from SI group and one from ExI group were took out from all analyses because they did not present *T. gondii* seroconversion. In the same set of experiments one mouse from ExI group was not evaluated as well, because it died 2 days before euthanasia (see scheme in Figure 1).

In the first set of experiments, immunohistochemistry (Figure 4) and qRT-PCR (Figure 5) analyses were carried out. No difference was found between SI and ExI groups for immunohistochemistry and qRT-PCR (*P* = 0.1250 and *P* = 0.7500, respectively). One sedentary infected mouse showed very little *T. gondii* DNA quantification (3 × less compared to second smallest quantification sample), but in concordance, in immunohistochemistry analysis, it showed the smallest (50% less compared to the mean of the group) brain cysts count among the group.

**Figure 2:**
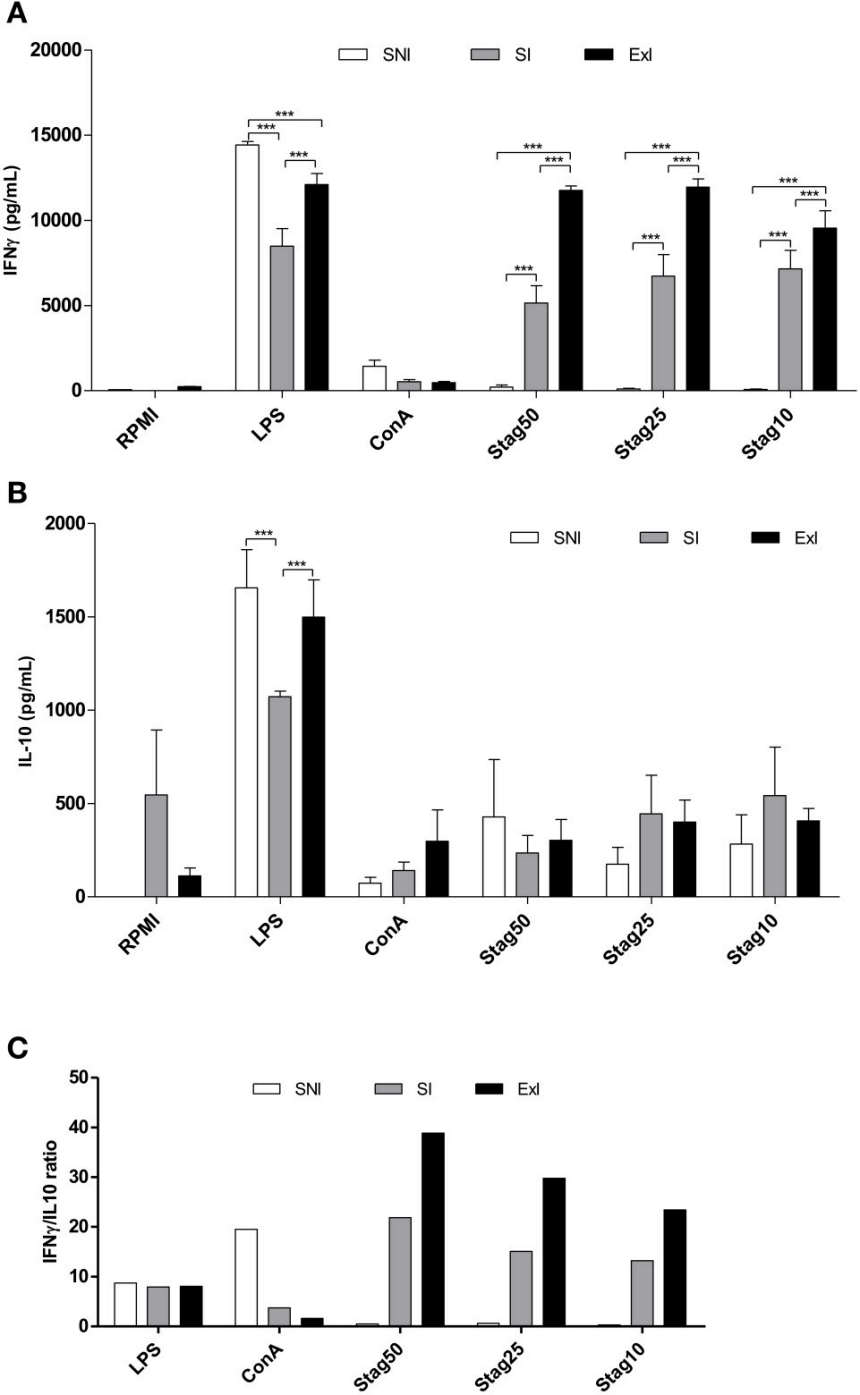
**IFN-γ (A)** and IL-10 **(B)** production from spleen cells from three mice groups from the first set of experiments. Spleen cells from the three groups (sedentary non-infected group, SNI; sedentary infected group, SI; and exercised infected group, ExI) were tested with five stimuli: bacterial lipopolysaccharide (LPS; 1 μg/ml), Concanavalin A (ConA; 10 ng/ml), *T. gondii*-soluble tachyzoite antigen (STAg; 50, 25, and 10 μg/ml). Values are indicated as mean ± SEM. ^***^P = 0.0006 and ^***^P = 0.0005 for IFN-γ and IL-10 measurements, respectively. **(C)** The IFN-/IL-10 ratios from all stimuli are also shown.

The Figures 2A,B, from the first set of experiments, shows IFN-γ and IL-10 production from splenocytes among three groups (SNI, SI, and ExI), respectively. For IFN-γ, statistical analysis showed differences among all groups and stimuli, when compared with RPMI and ConA stimuli (*P* = 0.0006). For LPS, control group (SNI) showed the highest IFN-γ production and the SI had the lowest. For all STAg concentrations (50, 25, and 10 μg/ml), ExI group had the strongest IFN-γ response. As expected, the control group (SNI) did not show significant amounts of IFN-γ production. For IL-10, it was observed significant differences only for LPS (SNI × SI and ExI × SI; *P* = 0.0005). As shown in Figure 2C, the IFN-γ/IL-10 ratios showed that ExI had the highest values, when the stimulus was STAg and the control group showed the highest when the stimulus was ConA.

**Figure 3:**
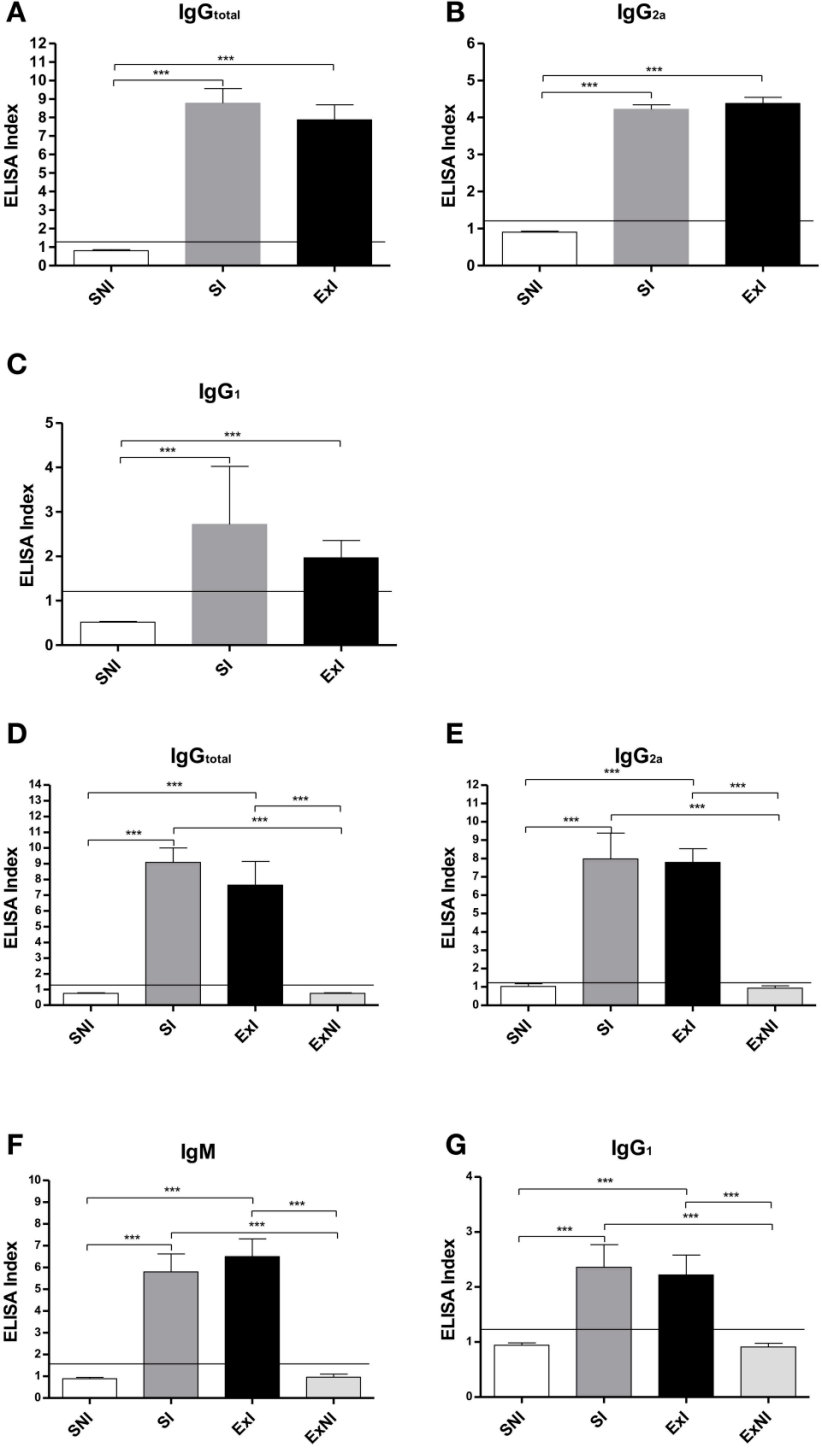
**Seroconversion assay [ELISA; first set of experiments (A–C)** and second **(D–G)]**. All the groups were tested before (data not shown) and 30 days after infection. Groups: sedentary infected (SI); Exercised Infected (ExI); Control groups [Sedentary non-infected (SNI) and Exercised—non-infected (ExNI)]. Cutoff was considered to be 1.2. Data express in mean ± *SD*. ^***^P < 0.001.

**Figure 4:**
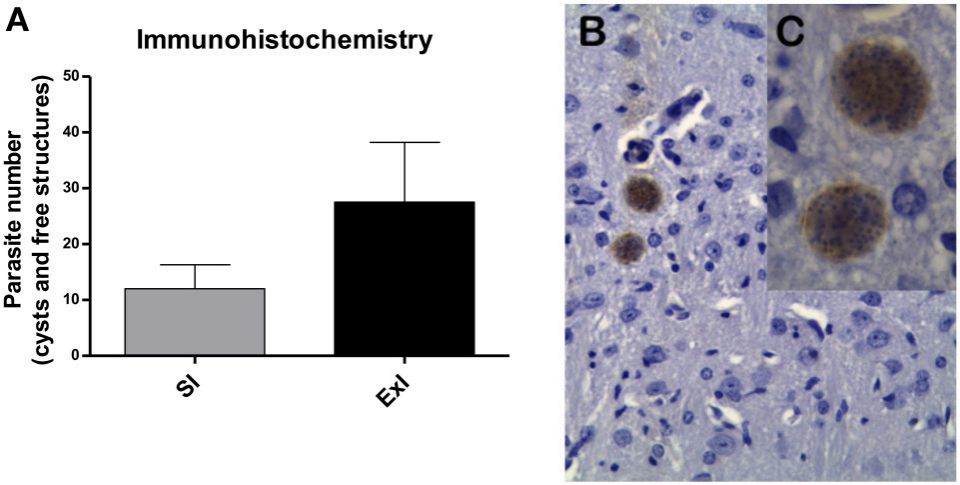
**(A)** Parasite and parasite-like structures cysts number in brain (first set of experiments). The number of cysts detected by immunohistochemistry in brain (half of the brain) of *T. gondii*-infected (ME49 stain) C57BL/6 mice in two groups: sedentary infected group (SI) and exercised infected group (ExI). No significant difference was found (*P* = 0.1250). Data are represented in mean ± *SD* of all values of independent dop. **(B,C)** Cysts images of 40 × and 100 × magnification, respectively.

**Figure 5:**
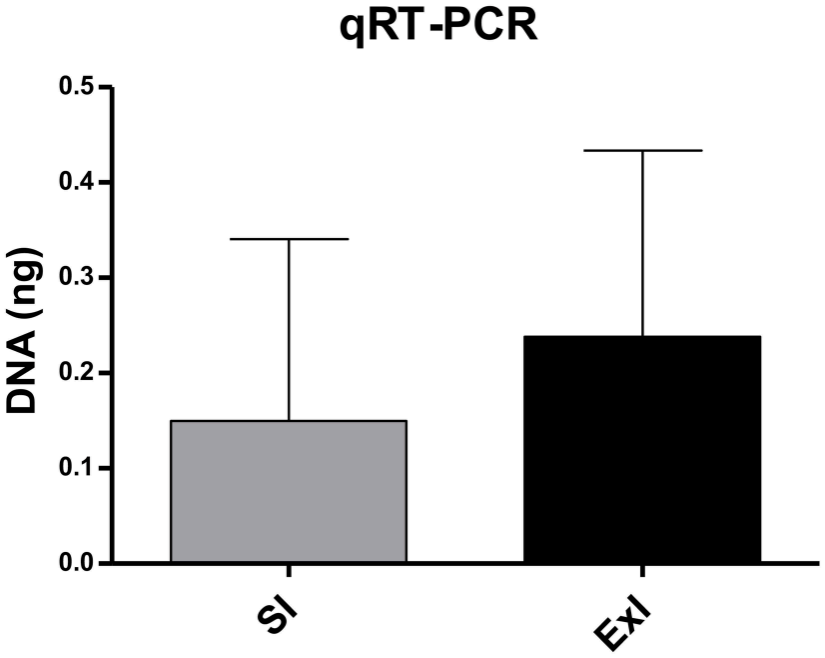
**qRT-PCR analysis of the first set of experiments**. Quantification of *T. gondii* DNA (ng) of sedentary infected group (SI) and exercised infected group (ExI). No significant difference was found (*P* = 0.7500). Data express in mean ± *SD*.

**Figure 6:**
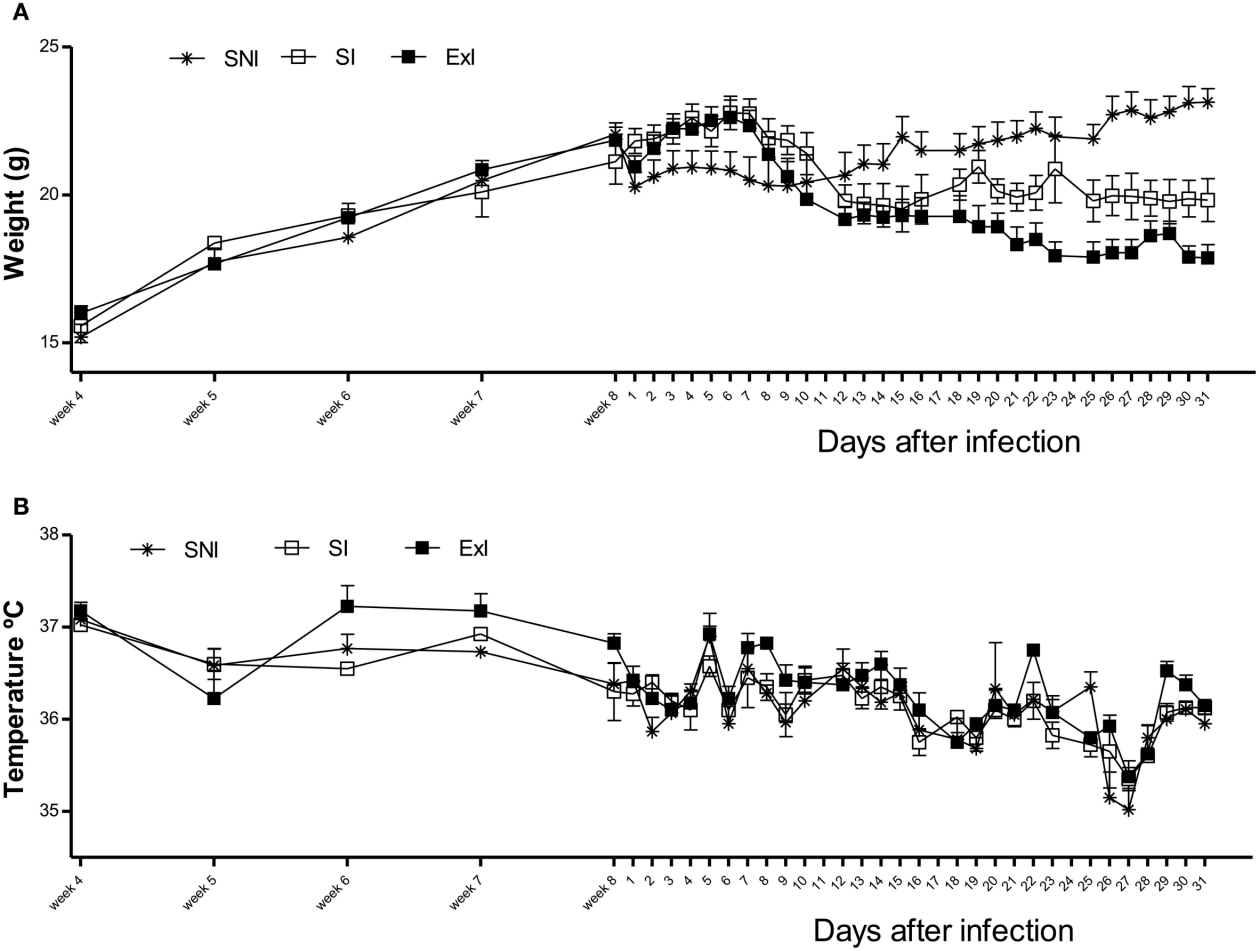
**Weight and temperature from the first set of experiments**. There were three groups, sedentary non-infected (SNI, control group), sedentary infected (SI), and exercised infected (ExI). **(A)** concerning the weight, there were no differences till the infection (*P* = 0.4682). After it (week 8), there were differences among the control and infected groups, and after day 23, there were differences between the infected groups too (*P* = 0.0345). **(B)** There were no significant differences concerning temperature measurements (*P* = 0.7650).

**Figure 7:**
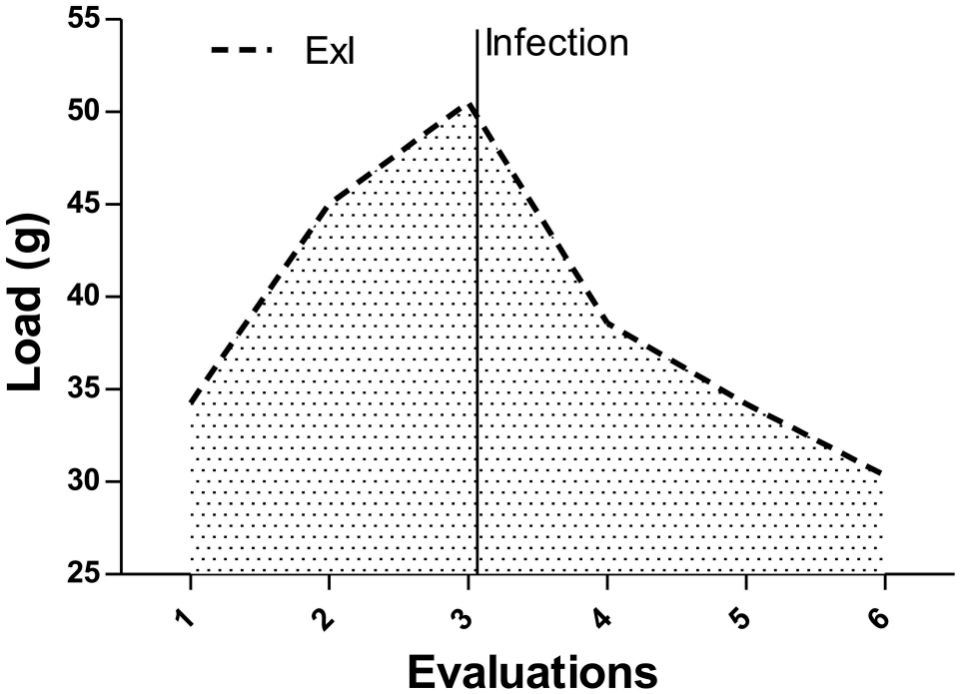
**Resistance exercise evaluation from the first set of experiments**. 1-RM evaluations of the Exercised infected group (ExI).

Weight and temperature measurements from the first set of experiments are shown in Figures 6A,B. Concerning the weight, no difference was observed till the infection (*P* = 0.4682). After it, weight showed differences among the control compared to infected groups, and after 23 days the infected groups also were found to be different, having the ExI group the lowest mean (Figure 6A; *P* = 0.0345). Concerning temperature, there was no significant differences among the groups (Figure 6B; *P* = 0.7650). The 1-RM resistance exercise evaluations for the Exercised infected group (ExI) in the first set of experiments were carried out as shown in Figure 7.

**Figure 8:**
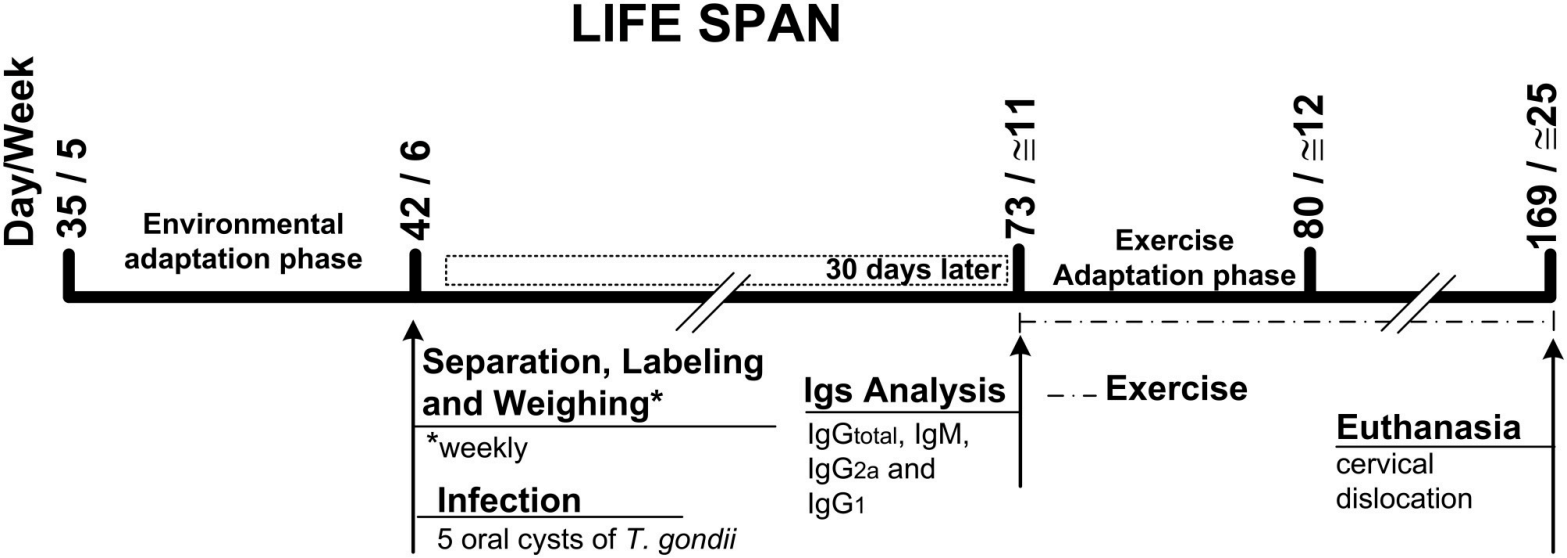
**Scheme of life span of the second set of experiments**.

**Figure 9:**
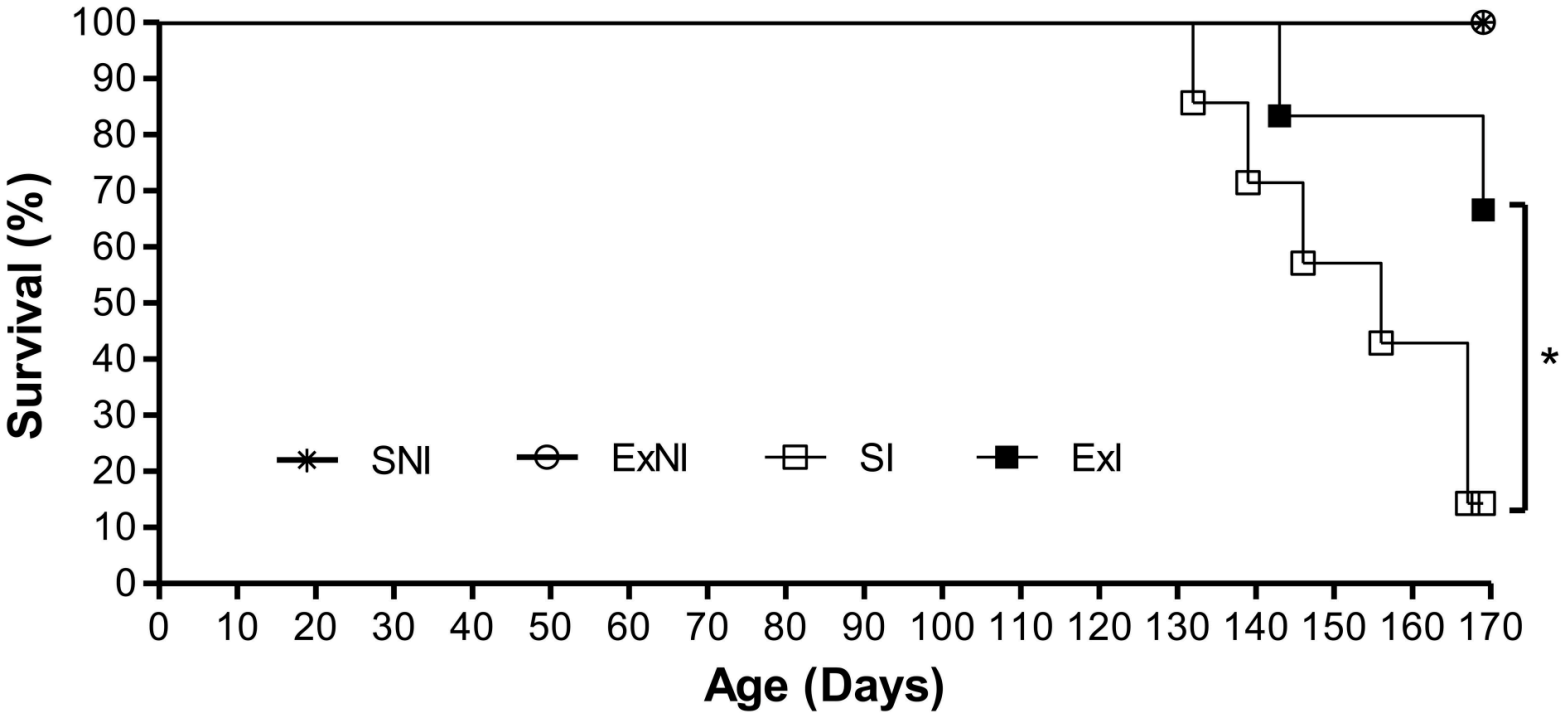
**Survival curve of C57BL/6 mice infected with ***Toxoplasma gondii*** (first set of experiments)**. Four groups: Non-infected sedentary (SNI; *n* = 6); Exercised non-infected (ExNI; *n* = 6); Sedentary infected (SI; *n* = 7); Exercised Infected (ExI; *n* = 6); Mice were infected with 5 cysts at age of 6th weeks. ^*^P = 0.0005 (Log-rank test).

**Figure 10:**
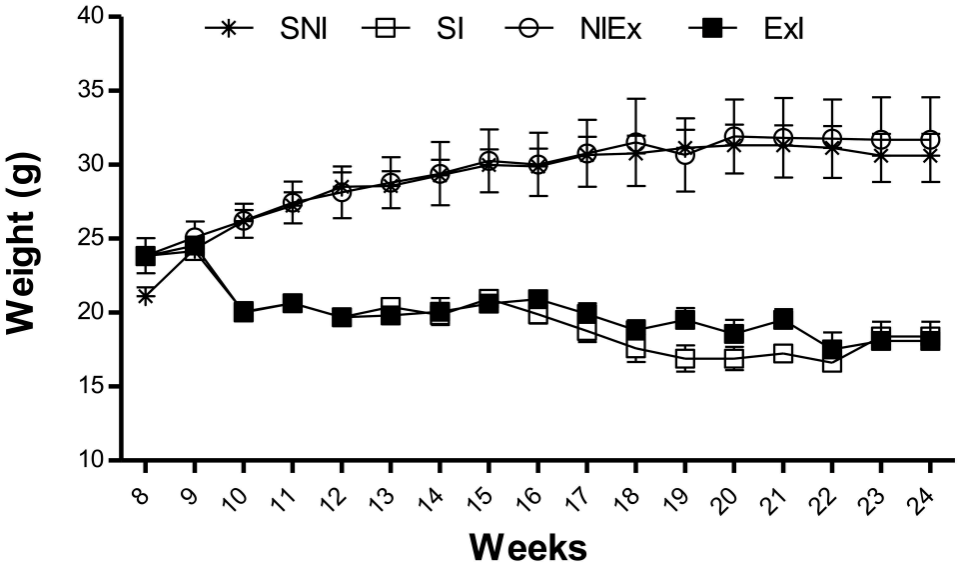
**Weight measurements from 8th week (just before infection) to the end of the second set of experiments**. Significant difference was found among the infected groups (ExI × SI) and non-infected ones (ExNI × SNI) (*P* = 0.0285), but there were no significant differences among ExI and SI or ExNI and SNI (*P* = 0.7685 and *P* = 0.7122). Data express in mean ± *SD*.

Concerning the survival rates of the animals (Figure 9), it was found a significant difference between exercised infected group (ExI) compared to sedentary infected group, as the exercised group survived longer than sedentary one (*P* = 0.0005). The first sedentary animal died 60 days after infection (day 132), and before the first animal from the exercise group died (day 143), one more sedentary mouse died (day 139). The second exercised animal died at day 169, but more four sedentary animals died before it (respectively, days 146, 156, 167, and 167). Once there was only one sedentary left, this set of experiments was considered to reach its aim and the mice were euthanized. The sedentary mouse that left showed signals to be recovering, once it reached 16.6 g and its last weight was 18.4 g, which was almost the same of 4 weeks earlier (18.9 g, 12th week; see Figure 10). Besides, its coat became sleeker, as it was before infection. Among the four exercised mice, two were weighing around 16 g and one was over 17 g. Their weights were decreasing and their coats were ruffled. The fourth mouse reached 19.4 g (3rd week after infection or 10th week in Figure 10) and had ups and downs during the experiment, but in the end it had a stable weight (±22 g) with glossy coat just as before infection. This mouse and the one over 17 g were running and practicing strength exercise regularly. The remaining animals could not run and/or practice strength exercise fully at least 10 days before euthanized them. As shown in Figure 10, there were significant weight differences among the infected groups (ExI and SI) compared to controls (ExNI and SNI; *P* = 0.0285).

**Figure 11:**
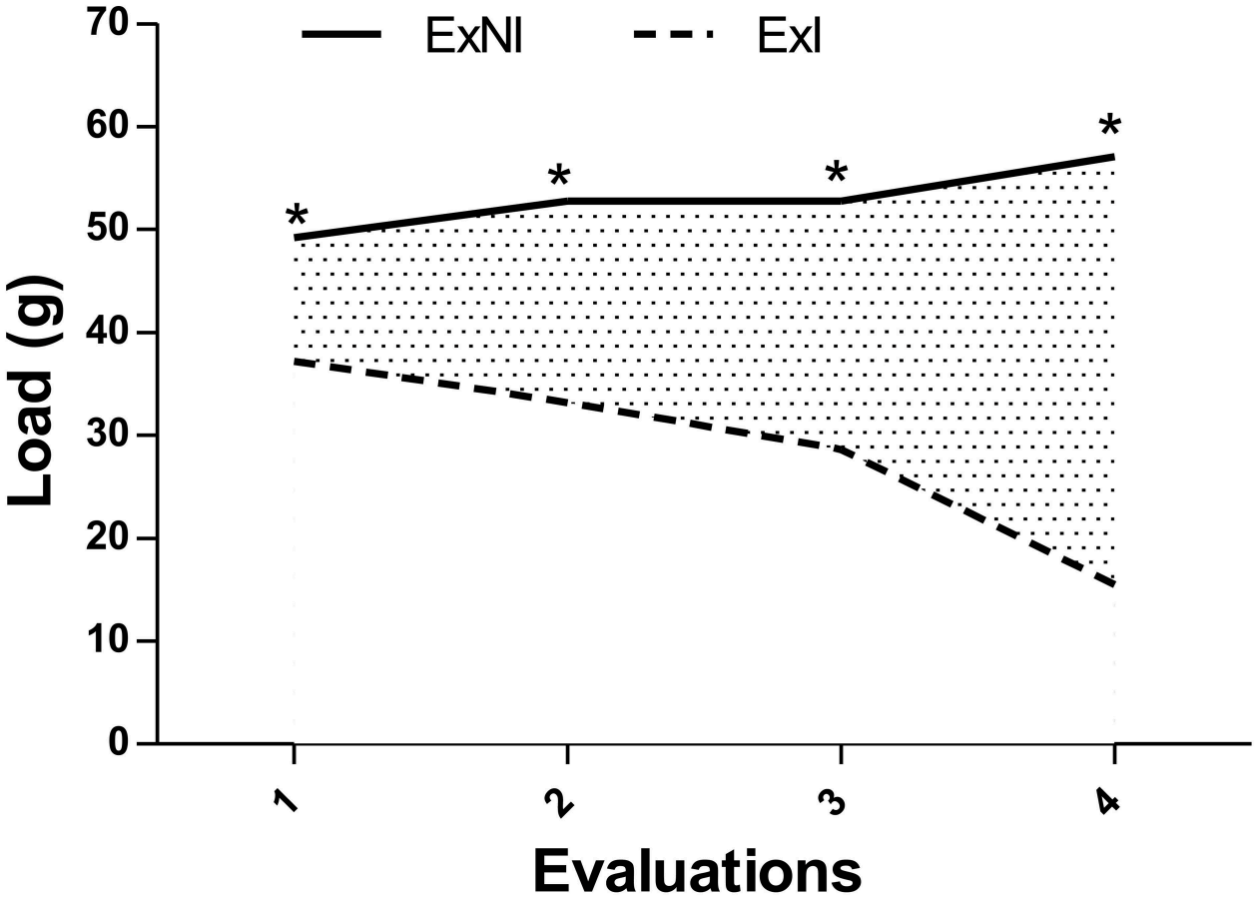
**Resistance exercise evaluation from the second set of experiments**. 1-RM evaluations of the Exercised infected group (ExI) and non-infected group (ExNI). All the means between groups were different ^*^P = 0.0008.

Before the death of the first exercised-infected mouse, four strength exercise evaluations were performed with the animals (Figure 11). The load mean of the first evaluation was statistically different between the groups, ExI (37.2 g) and ExNI (49.2 ± 3.18). This difference became higher till the end of the evaluation cycles (15.48 ± 15.09; 57.08 ± 5.26, respectively, *P* = 0.0008).

**Figure 12:**
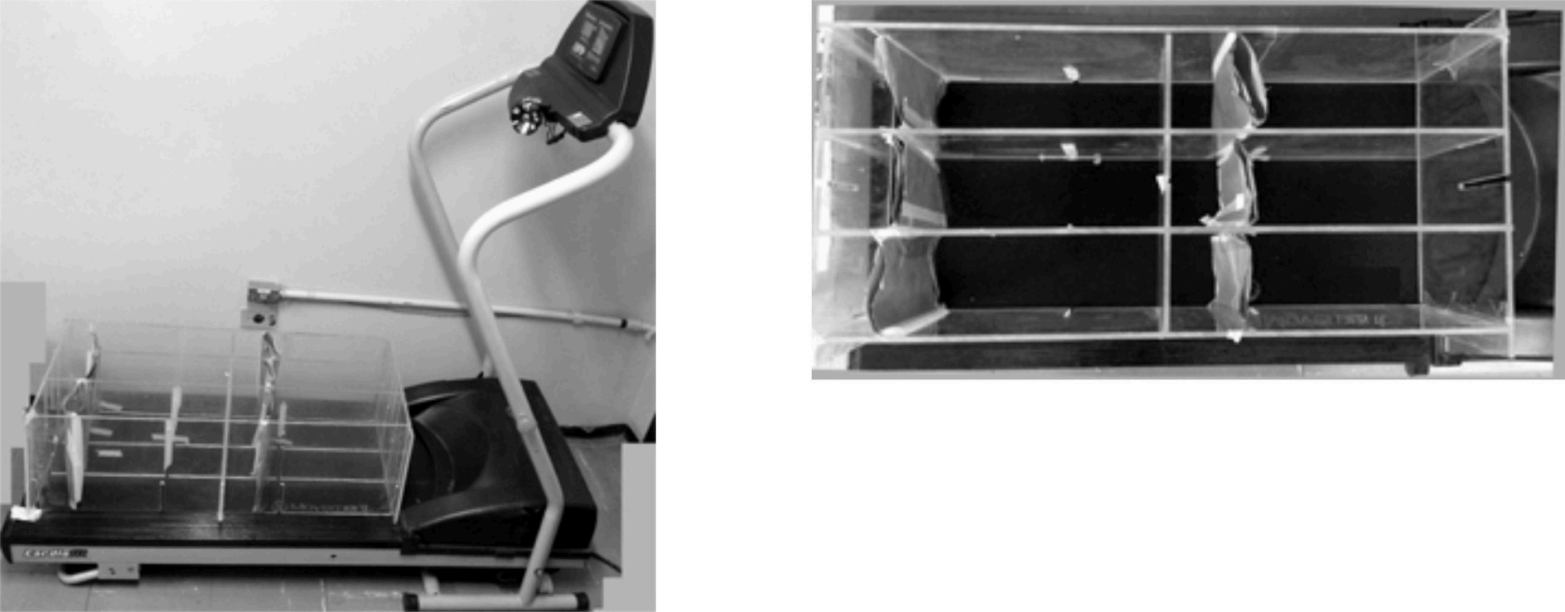
**Treadmill adapted for mice running speed and added acrylic cages**.

**Figure 13:**
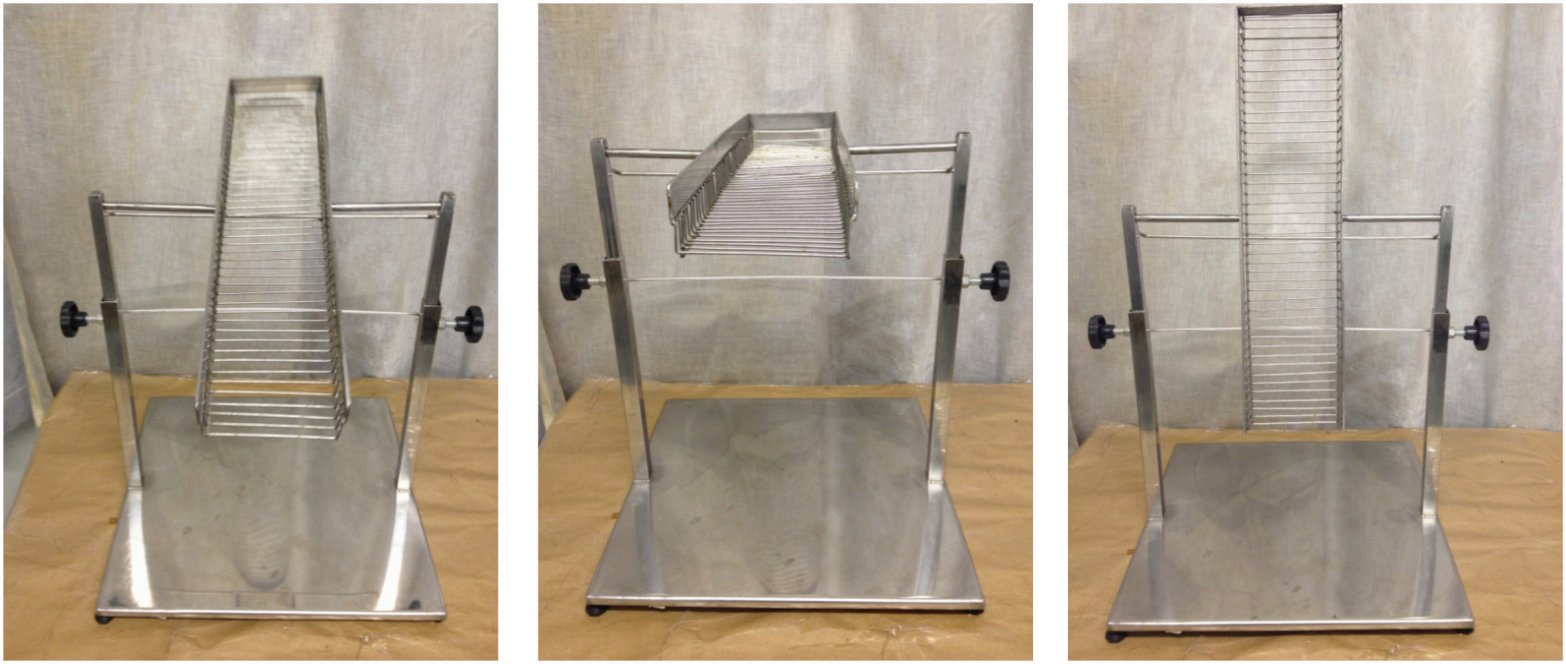
**Climbing apparatus in different positions**.

**Figure 14:**
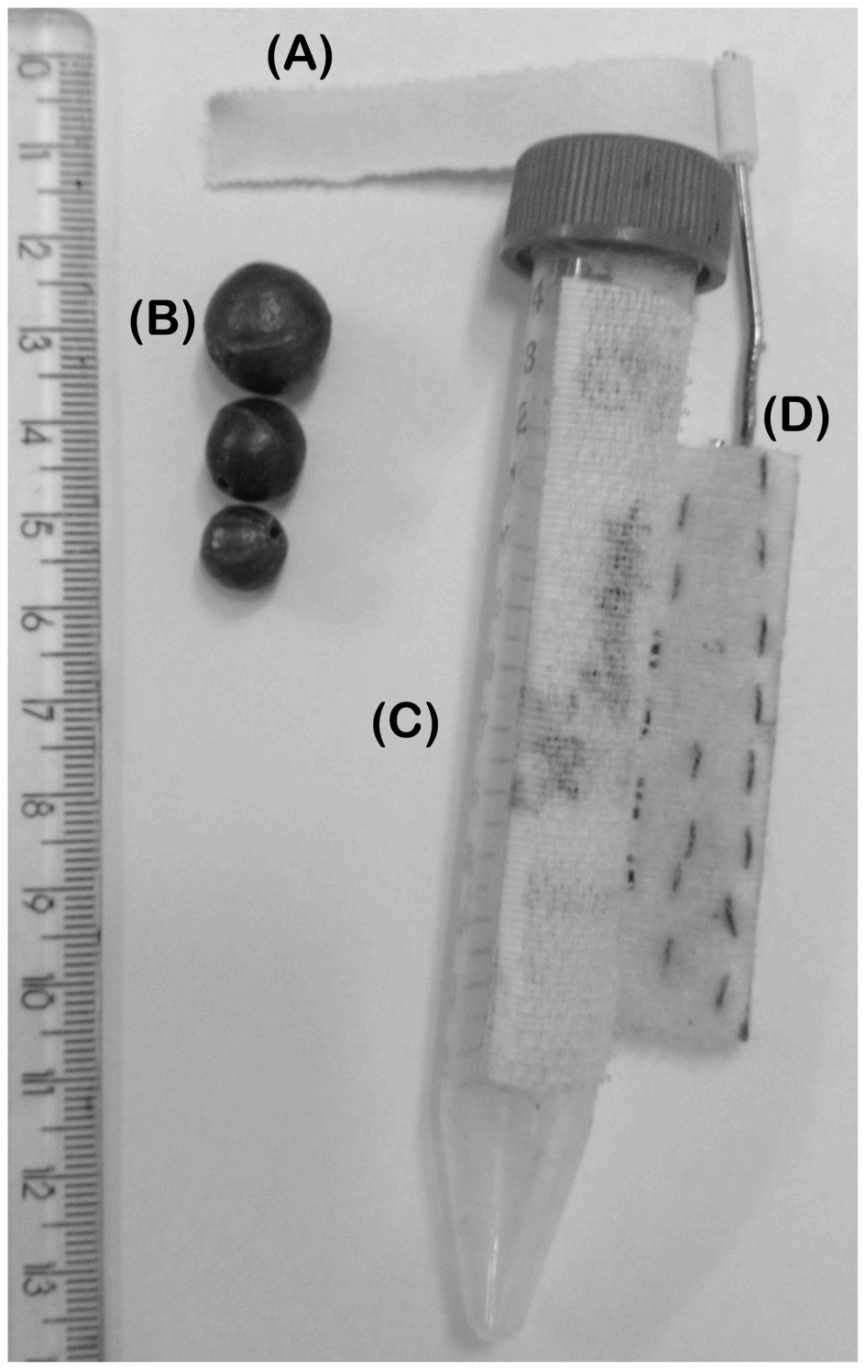
**Apparatus that allows one to control the load for resistance exercise**. It is shown from the left to the right the following items: **(A)** adhesive tape that is fixed to the mouse's tail and to item “D”; **(B)** lead balls that are used to load the item “C”; **(C)** 15 mL “Falcon” tube with Velcro (hooks) posted in side that is used as a loader of item “B” and to be fixed at item “D”; **(D)** Velcro (loops) posted to a metal rod with adhesive tape.

## Discussion

In the present study, the first central finding was concerning the determination of IFN-γ and IL-10 profiles, clearly showing that exercised mice had the strongest Th1 response under STAg stimulation by presenting the highest IFN-γ production and IFN-γ/IL-10 ratio. These results are in agreement with those described by Terra and colleagues demonstrating that lymphocytes and macrophages from exercised mice have enhanced their efficiency to produce high pro-inflammatory cytokines (e.g., IFN-γ, IL-12, TNF-α) when they were *in vitro* stimulated with LPS, ConA, and/or *Leishmania major* antigen (Terra et al., 2013). In the present study, beyond the data concerning STAg stimulation, when the splenocytes from ExI group were stimulated with LPS and ConA, the responses were not very strong for LPS and weak for ConA. In consonance with Terra and colleagues previous physical exercise can lead the host to a more efficient IFN-γ response, resulting in a better Th1/Th2 balance when challenged by an intracellular parasite (Terra et al., 2013), as *T. gondii*.

Additional finding from the present study was that exercise mice infected by *T. gondii* lived longer than sedentary-infected ones, taking into account that mice performed both aerobic and strength exercise regularly in our experimental design. It has already been described that muscle can be considered as an endocrine organ by producing significant amounts of cytokines during strength or aerobic exercises, as IL-6 (Pedersen and Febbraio, 2008). IL-15 can also be produced by muscle *in vitro* (Grabstein et al., 1994) or after strength exercise in humans (Nielsen et al., 2007), as well IL-7 (Haugen et al., 2010). IL-7 plays an important role to provide signal to naïve and memory T CD8^+^ cell survival and IL-15 is believed to be crucial to direct the basal proliferation of memory T CD8^+^ cells (Ma et al., 2006). When IL-7 was added to human T cell cultured *in vitro*, it was observed an upregulation of IFN-γ expression in a dependent-dose manner (Borger et al., 1996). CD8^+^ T cells and IFN-γ are essential effectors to mediate resistance to acute and chronic *T. gondii* infection (Gazzinelli et al., 1991, 1992). It has also been suggested that these findings have important implications to understand the use of IL-7 and IL-15 as adjuvant for therapeutic vaccination protocols against intracellular pathogens, considering that CD8^+^ T cells constitute a critical component of protective immunity (Bhadra et al., 2010). Recently, previous regular physical exercise has provided to be an adjuvant in vaccination (Bachi et al., 2013). Besides, it can improve the life quality during the aging process and extend life span (Handschin and Spiegelman, 2008). Romeo et al. have summarized the main factors that could affect the infection risk in humans, by describing that, while aging and sedentary habit boost the infection risk by increasing immunodepression, moderate but regular physical activity acts fighting immunodepression and decreasing the infection risk (Romeo et al., 2010). Based on these results, we hypothesized that, by exercising regularly, specially strength one, the host could improve its adaptive immune response against *T gondii* by producing appropriate IFN-γ levels, being this event critical to increase host survival. Future studies should address whether this feature comprises activation of CD8^+^ T cells as the major protective mechanism in this experimental design.

Regarding our data obtained from ExI group in the second set of experiments, the review from Walsh et al. discussed that physical exercise somehow can increase the gastrointestinal permeability, but it could happen only after exhaustive exercise (Walsh et al., 2011a). Besides, the mortality in C57BL/6 mice after peroral infection appears to be due to severe necrosis of the small intestine, which has been shown to be CD4+ T cell-dependent and IFN-γ mediated (Liesenfeld et al., 1996). Chao et al. found that swimming exercise is not deleterious to mice acutely infected with *T. gondii* Me49 (Chao et al., 1992). On the other hand, our second set of experiments, when the mice performed physical exercise only 30 days after infection and only 5 cysts were used, it showed that exercised mice survival longer than sedentary ones. Taking these data together, physical exercise may potentiate the parasite damage in acute phase. To further elucidate this matter, future studies involving physical exercise in acute phase of *T. gondii* infection should be carried out, especially regarding its intensity and type (e.g., strength and/or aerobic).

Concerning 1-RM evaluation in the second set of experiments, our results showed that both exercised group (ExI and ExNI) were different (*P* < 0.001) and this difference became higher after 10 weeks of observation. These data indicate that infection can interfere in exercise performance, even when exercise started in chronic phase of *T. gondii* infection. In agreement with these data, we also demonstrated in the present study that the 1-RM evaluations of the ExI group from the first set of experiments, which points that 1-RM increased till the infection, reached more than 200% of their body weight, and decreased dramatically to the lowest value after it.

In the present study it was chosen voluntary running instead forced one (e.g., electrical stimulation). Lerman and colleagues found among groups of six mice that C57BL/6J consistently showed the highest level of voluntary wheel-running performance and the lowest when the exercise was forced (Lerman et al., 2002). In our study, it was carried out an incremental maximal speed test during 6 weeks, when the mice reached maximal speed of 20 m/min, 1% inclination (data not shown). These data were similar to those found previously (18.6 ± 6.1 m/min first 2 weeks of training and 24 m/min after it; Lerman et al., 2002). Lerman and colleagues used in their experiments older mice than it was done in ours. In our first set of experiments, mice were 4–13 weeks old, whereas the voluntary running experiment was carried out using mice aging 5–6 month-old in their study. In our second set of experiments, mice started to exercise with 2.5 months old and finished with 5.6 month-old. As previously seen in our work, one exercised group in each set of experiments were infected by *T. gondii*. Regarding these data and, once the present work also aimed to maintain the mice active and to avoid injures, the safe speed considered was 14 m/min. The lactate analysis was avoided concerning the stressor factor that regular collection of blood could cause to the animals.

To the best of our knowledge, only Chao et al. have worked so far with *T. gondii* infection and exercise, and their results showed that, although the infection caused a significant elevation of serum TNF-α levels, it was attenuated by a daily swimming program (Chao et al., 1992). It is important to emphasize that they have worked with female BALB/c mice and the infection route was intraperitoneal (IP). In our study, we used C57BL/6 male mice and the infection route was peroral, which is the more frequent route of *T. gondii* natural infection. Mouse models, especially from C57BL/6 lineage, have been extensively studied in the last two decades and it has been shown to be advantageous because their availability and facility to generate genetically engineered and mutant lineages, allowing to investigate the molecular mechanisms behind aging related muscle wasting (Ballak et al., 2014). Concerning *T. gondii* infection, the C57BL/6 lineage model stands out due its susceptibly to this parasite (McLeod et al., 1984). Henry and Beverley first demonstrated the existence of gender differences in terms of susceptibility to *T. gondii* infection, being females less susceptible to this infection than males (Henry and Beverley, 1976). Once testosterone leads the animal to an anti-inflammatory profile (Pinzan et al., 2010), it could be an explanation for increasing the host susceptibility to protozoan parasites (Roberts et al., 2001). Meyer et al. investigate the influence of the infection route in the severity of experimental infection by *T. gondii* and they concluded that, although intraperitoneal route can guarantee the infection, it can turn the parasite burden faster when compared to peroral one, which can lead to a more severe infection and lead the host to succumb more quickly, either by affecting parasite replication or expansion of human inflammatory cells (Meyer et al., 2013). These authors also suggested that the peroral infection can be useful for more in-depth analysis of *in vivo* immune responses against *T. gondii*, as well as for therapeutic studies of candidate drugs for treatment (Meyer et al., 2013). Currently, it is remarkable that physical exercise has been used to prevent and to treat many diseases, as a non-pharmacological therapy *in vivo* studies (Walsh et al., 2011a,b). Thus, taking these data together, it is suggested that future studies regarding experimental model of physical exercise and *T. gondii* infection might be set up using the animal genotype and infection route as described in the present study.

In accordance to the World Health Organization which recommends several practices of regular physical activities, and muscle-strengthening for humans older than 18-years-old (World Health Organization, 2010), the present study used both aerobic and strength exercise. Sedentary and obesity, in early senescence, are very important risk factors leading to many different diseases (Handschin and Spiegelman, 2008). Thus, future experimental studies should be conducted approaching parasite infection and obesity in elderly mice models looking forward to observe simultaneously more than one physical capacity (e.g., aerobic and strength). These future studies could drive us to a whole new scenario for a better understanding concerning how regular physical exercise might influence immunological system in seniors, obese or not, infected by parasites, specially by *T. gondii*.

In summary, it can be pointed out two central findings described in the present study, which showed a strong positive data regarding extending life span of exercised infected mice, and the enhancement of the splenocytes activation expressed by producing of higher levels of IFN-γ leading to a higher IFN-γ/IL-10 ratio, when compared to sedentary infected mice. Therefore, these findings can be considered strong evidences demonstrating that previous regular exercise training can stimulate immune response against deleterious effects of *T. gondii* infection. Future works underlining the effects of strength exercise alone and together with aerobic one involving IL-7, IL-15 production, as well as CD8^+^ T cell activation toward infection in a model of experimental toxoplasmosis, could give new insights regarding the effective relevance of each type of exercise has to decrease morbidity and mortality rates due to *T. gondii* infection.

## Ethics statement

All experiments and procedures were conducted according to institutional guidelines and approved by the Ethical Committee in Animal Experimentation (CEUA-UFU Protocol No. 053/10). The physical exercises were conducted in the dark circle from 07:00 p.m. to 10:00 p.m. The animals had previous 1 week adaptation in experimental room and they were kept under standard conditions, natural photo-period, in a temperature-controlled room (25oC), with *ad libitum* food and water intake, in the Bioterism Center and Animal Experimentation, Federal University of Uberlândia, Brazil.

## Author contributions

MB was involved all procedures of this work, as mouse exercising and infection, cytokine and antibody assays, statistical analysis, determination of brain parasite load for qPCR, and preparation of the draft manuscript. MS participated in assay, mouse infection procedures and cytokine and antibody assays. FA and LM were responsible to animal care and exercising procedures. FC participated in the immunoassays to determine the levels of immunoglobulin isotypes. LC was involved in experiments carried out to determine parasite burden. NS was responsible to perform the immunohistochemistry assays. NP participated in the building process of the physical exercise apparatus and in the result discussions. TM and JM were involved in the experimental design, data analysis, and revision of the manuscript. All authors read and approved the manuscript.

### Conflict of interest statement

The authors declare that the research was conducted in the absence of any commercial or financial relationships that could be construed as a potential conflict of interest.
